# Weight-bearing MRI with a knee flexion angle of 20°: a study on additional MRI investigation modalities to support a more accurate understanding of patellofemoral instability

**DOI:** 10.1186/s12891-021-04733-4

**Published:** 2021-10-25

**Authors:** J. Leiprecht, F. Mauch, J. Huth, L. P. Ambros, R. Best

**Affiliations:** 1 Department of Orthopaedics, University of Ulm, Oberer Eselberg 45, 89081 Ulm, Germany; 2 Department of Orthopaedics, Sportklinik Stuttgart GmbH, Taubenheimstraße 8, 70372 Stuttgart, Germany; 3 Department of Surgery and Orthopaedics, St. Vinzenz Hospital, Kirchenweg 15, 87459 Pfronten, Germany; 4 Department of Orthopedic Sports Medicine, University of Tuebingen, Hoppe Seyler Strasse 6, 72076 Tuebingen, Germany

**Keywords:** Patellofemoral instability, Magnetic resonance imaging, Weight-bearing, Knee flexion angle, Risk indices

## Abstract

**Background:**

Diagnosing patellofemoral instability disorders correctly, weight-bearing MRI (WB-MRI) has become an option. Aiming for a best possible accuracy in displaying potentially underlying causes, the named MRI modalities were sporadically even investigated in different knee flexion angles. However, despite confirmed MRI-outcome-differences between WB-MRI and non-WB-MRI, none of the described MRI modalities have so far established themselves. Mainly this is due to an unfeasibility in daily clinical routine in regard to time and economic aspects. Thus, we intended to evaluate an additional but reduced patellofemoral MR-imaging solely in a relevant 20° of knee flexion under WB- and non-WB-MRI conditions.

**Methods:**

Seventy-three subjects with and without patellofemoral instability were investigated under supine as well as under WB-MRI conditions in a 20° of knee flexion angle. Patellofemoral risk indices in the sagittal plane (Insall-Salvati-Index, Caton-Deschamps-Index, Patellotrochlear Index) and the axial plane (Patella tilt of Fulkerson and Sasaki) were detected and compared between the different MRI conditions. Significance, reliability and Cohen’s effect size was calculated.

**Results:**

Nearly all assessed indices showed significant differences between patients and controls in the different MRI positions. Comparing pairwise, all measured indices failed to show significant differences between the two MRI positions. However, patella tilt angles of the patient group showed an elevation from supine to WB-MRI (14.00 ± 7.54° to 15.97 ± 9.10° and 16.34 ± 7.84° to 18.54 ± 9.43°). Here, Cohen’s d showed small to medium effects between supine and WB-MRI.

**Conclusion:**

In comparison to standard MRI in supine position, axial risk indices seem to be accentuated under WB-MRI and a knee flexion angle of 20°. In particular, symptomatic cases with inconspicuous conventional MRI imaging, additional MRI imaging only in the axial plane in a 20° of knee flexion could be beneficious and useful in clinical daily routine.

## Background

Anterior knee pain, patellar dislocation and chronic patellofemoral instability are counted among the most common pathologies of the knee especially in adolescents and young adults [1–4]. About 50% of affected patients are diagnosed with patellofemoral maltracking [2] meaning a functional and/or structural mismatch between patellar and trochlear joint partners. Here, excessive patellar lateralization and or tilting in relation to the femur are most commonly observed especially during activation of the quadriceps muscle [2].

Currently, Magnetic Resonance Imaging (MRI) of the extended knee in supine and non-weight-bearing condition plays a decisive role in assessing underlying causes of the patellofemoral disbalance in clinical daily routine [2, 5]. On the basis of mainly axial and sagittal MRI planes many geometric threshold values (e.g. patellar height, axial patellar tilt angle etc.) have been determined [6–9] to enable a best possible accurate estimation and diagnosis of patellar maltracking [6–9]. Furthermore, guidelines have been established to facilitate the choice of subsequent optimal conservative and operative therapeutic options.

In the past decade the current MRI routine has however been under dispute [2, 4, 6, 7] as it might not reflect the individual complexity of a patient’s realistic patellofemoral motion behavior under loaded conditions [10]. Most patients suffer from patellofemoral instability and pain only during active transition from extension to early knee flexion (20°-30°) while performing normal activities [2, 4, 11]. Justifiably so, doubt exists that non-weightbearing-MRI (NWB-MRI) of an extended knee “in rest” with an inactivated quadriceps muscle is able at all to properly display the potentially problematic excessive patellar motion.

Additionally, previous biomechanical studies [12] show that the greatest tendency towards patellar lateralization was not found in full extension but in an active 20° flexion angle of the knee.

In order to address this issue, a possible approach in the course of investigating patellofemoral maltracking could be to perform a weightbearing MRI (WB-MRI) during upright positioning of the patient [1, 2, 4, 10, 13]. Some even suggest the performance of MR-imaging in different flexion angles of the observed knee [1]. Here it has been confirmed that MR-imaging solely conducted in full extension of the knee might over- or underestimate patellofemoral imbalance [1, 2]. This is supported by the finding that more than 20% of patients suffering from patellofemoral imbalance showed no pathologies in the standard MRI in supine position whereas “abnormal” patellar motion was detected in these patients upon performing a weight-bearing MRI [4].

However, especially due to limited practicability of WB-MRI in daily routine and despite revealing the named deviating results presented in a scientific set up [1, 2, 8, 14] none of the described MRI methods has established itself so far. Also, a clear consensus towards particular additional standard WB MRI protocols in order to facilitate the implementation of WB-MRI in daily routine has not yet been reached.

Hence, the aim of the present study was to investigate the patellofemoral joint under weight-bearing and non-weight-bearing MRI examination concerning differences regarding the detection of relevant risk factors in patellofemoral joint-instability. In light of common routine, we intended for the “additional” weight-bearing modalities to meet the criteria of a practical setup, instead of the performance of a real time MRI [2] or the consideration of different flexion angles [1]. Therefore, based on the described biomechanical observations [2, 4, 6, 7, 11], one single “early” flexion angle of the knee was chosen.

We hypothesized that the results of the measurement of patellofemoral instability under weightbearing MRI examined solely in a 20° knee flexion angle of the knee would accurately reveal pathologies as well as differ from the results of standard MRI in supine positioning of the patient in terms of significance and effect size.

## Methods

In an explorative case-control-study 73 subjects were investigated for patellofemoral instability risk factors using MRI. Hereof, 35 patients (PG: patient group) had experienced at least one intrinsic (no external impact) patellar dislocation and presented to our clinic in order to be scheduled for patellofemoral realignment surgery.

A control group (CG) consisted of 38 healthy volunteers without any history of patellofemoral pain or instability.

Besides the occurrence of one or more patellar dislocations further inclusion requirements for the study were closed epiphyseal plates and an age above 16 years. Patients with a history of major traumatic incidents (e.g. knee distorsions), additional injuries (e.g. cartilage damage, non-patellofemoral ligamentous injuries (ACL, MCL etc.)) and previous surgeries of the relevant knee were excluded from the study. Prior to inclusion all patients gave their written informed consent for participation.

The patient group consisted of 23 female and 12 male subjects (mean age 25.3 years ±6.8 years, mean height 172.9 cm ± 9.9 cm), whereas the control group included 13 female and 25 male volunteers (mean age 29.8 years ±10.1 years, mean height 177.5 cm ± 5.7 cm).

### MRI imaging

For MR imaging, corresponding to previous studies [4], a dedicated, open MRI unit (G-scan-Esaote, 0.25 Tesla, Genoa, Italy) was used. All patients underwent a standardized protocol consisting of sagittal (TR 1700 ms, TE 75 ms, FOV 210 × 210, ST 4 mm) and axial (TR 1000 ms, TE 22 ms, FOV 210 × 200, ST 4 mm) T2 weighted fast spin-echo sequences in supine and upright weight bearing position.

Patients and controls were positioned on the MRI examination table with a clinically assessed 20° flexion angle of the knee by placement of a configurated foam roll underneath the examined knee. In accordance to the setup in previous studies [1], the examined legs were positioned in 15°-20° external rotation of the feet.

**Fig. 1:**
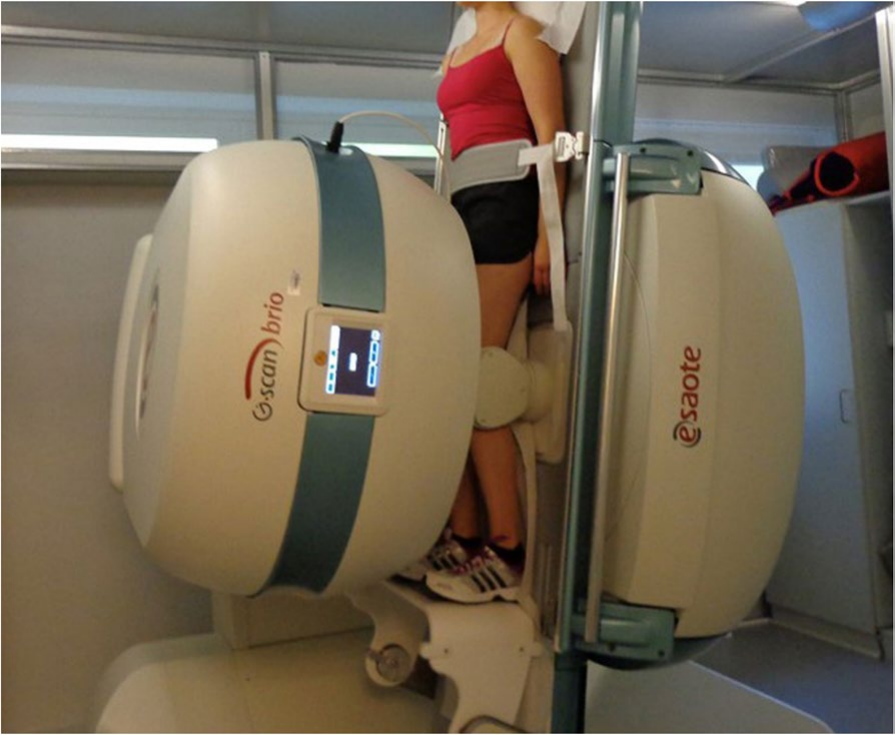
MR Imaging of the left knee in upright (81° tilt) position

Initially, the MRI unit was positioned in a vertical tilt of 81° for the upright weight bearing imaging. Due to the upright position with slightly flexed knees patients automatically contracted their quadriceps muscle reflecting a normal stance phase [Fig. 1].

**Fig. 2:**
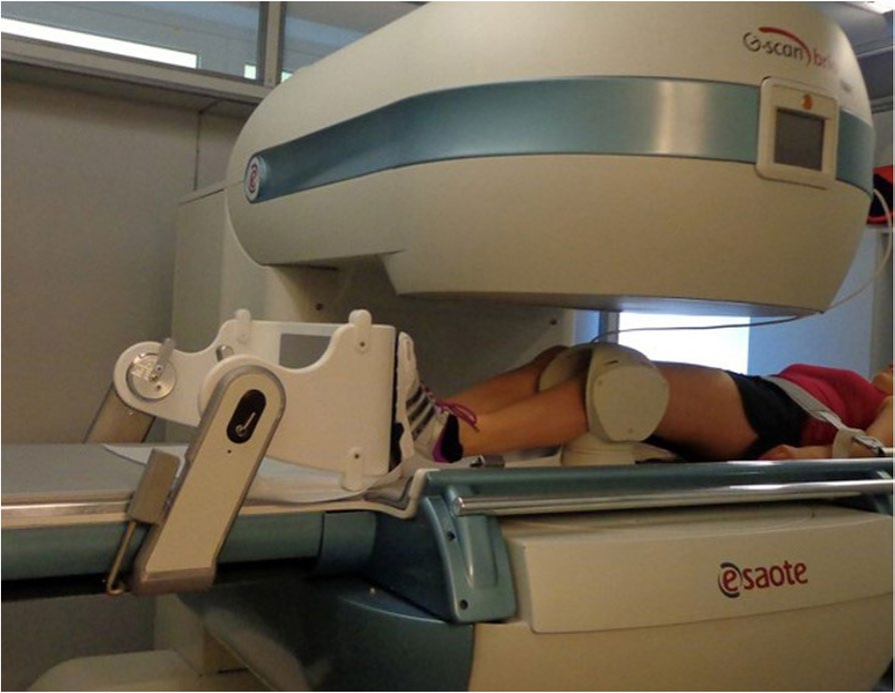
MR Imaging of the left knee supine position

Subsequently the unit was rotated to a horizontal tilt of 0° in order to perform the same imaging protocol in supine position [Fig. 2]. Here the positioning of the patient required no contraction of the quadriceps muscle and patients were additionally asked to relax the extensor muscle best they could.

During weight bearing imaging as well as at the beginning and at the end of the supine imaging an examiner was present in order to supervise correct positioning of the patient. The total duration of MR-imaging was 15:47 min. (Scout: 0:13 min.; Localizer: 0:38 min.; upright imaging sagittal T2 3:04 min.; axial PD 3:46 min.; Supine imaging sagittal T2 3:04 min.; axial T2 5:02 min.)

All obtained images were evaluated using meddix-VIEW/PRO software (Informatics Systemhaus GmbH & Co KG, Version 2.8.0.2012, Reversion 002R). Images Pictures were measured twice (t_1_ + t_2_) by one examiner (E_1_) within an interval of 8 weeks in order to detect the intra-observer reliability. A second independent second examiner (E_2_) repeated all measurements for inter-observer reliability (E_1_ + E_2_).

### Measured indices

**Fig. 3:**
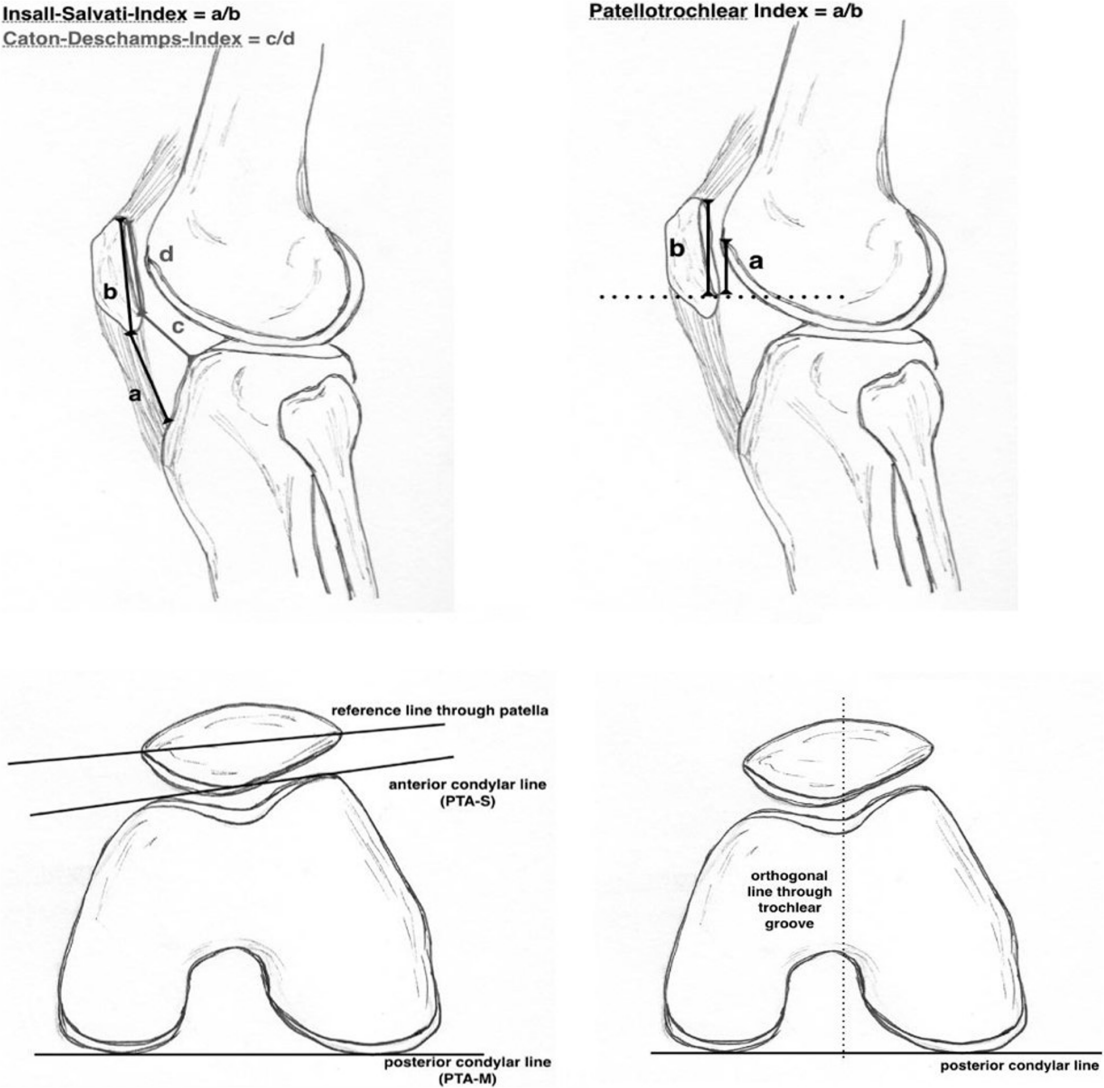
Schematic indices of patellofemoral joint anatomy (manually designed by first author LJ)

The measured indices and their schematic depiction are shown in Fig. 3.

For evaluating patellar height Insall-Salvati-Index (ISI), Caton-Deschamps-Index (CDI) and Patellotrochlear Index (PTI) were assessed.

The ISI is defined as the patellar tendon length relative to the maximal diagonal length of the patellar bone [15] and is therefore highly dependent on patellar morphology. Values lower than 0.8 are regarded as “patella baja”, whereas values above 1.2 are considered as “patella alta”.

The CDI is defined as ratio between the distance measured from from the distal end of the retropatellar cartilage to the tibial plateau and the sagittal length of the retropatellar cartilage [16]. Values above the threshold of 1.2 are determined as “patella alta”.

The PTI is defined as the ratio between the length of the trochlear cartilage overlapping the patella and the patellar cartilage length on the midsagittal MR image [17]. Values above 0.5 are considered as a “patella baja”, values beneath 0.125 a “patella alta”.

To evaluate the congruence of the patellofemoral joint in the axial plane, the patella tilt angle was measured according to Fulkerson [18] and Sasaki [19].

The patellar tilt angle (PTA) is determined as the mediolateral angle of the patella in the axial plane in relation to the femur. When determining the modified patella tilt angle by Fulkerson (PTA-M) a tangential line is drawn at the posterior condyles as a line of reference [18]. In comparison the patella tilt angle of Sasaki (PTA-S) is measured by placement of the tangential line at the anterior femoral condyles [19].

### Statistics

Statistical evaluation has been managed using the Statistic Software IMB SPSS Statistics Version 21.0. Differences between the single groups were assessed with the Mann-Whitney-U-test and Cohen’s effect size (d). The accuracy of indices measurements was set to two decimals. Value of significance has been stated at 5% (*p* <  0.05).

Post-hoc power analysis was performed with G*Power (freeware, version 3.1.97, developed by University of Duesseldorf, Germany). Power was calculated for significant values only and accepted as sufficient above 0.8.

For Cohen’s d, values of 0.2 were rated as small, values of 0.5 as medium and values of 0.8 as large effect [20, 21].

Inter-class-correlation coefficient (ICC) for inter- and intra observer reliability were calculated. An ICC > 0.75 was considered excellent, ICC > 0.40 and <  0.75 as fair to good, and ICC <  0.40 as poor [22].

The study was approved by the appropriate State Medical Board Ethics Committee (Ethik Kommission der Landesärztekammer Badenwürttemberg, No. F-2013-062).

## Results

**Fig. 4:**
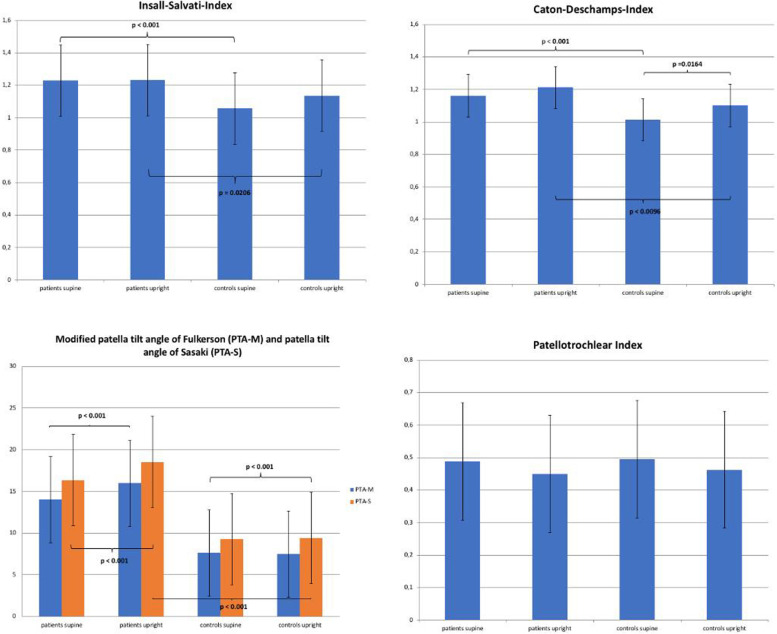
Bar chart of different risk factors of patellofemoral instability in patients and healthy control subjects in supine and weight-bearing MRI

All values and statistical comparisons between patients and controls as well as between supine and upright position are shown in Table 1. For better visualization, additionally the most relevant comparisons are displayed graphically (Fig. 4).

**Table 1:** Mean values, standard deviations (SD), *p*-values and cohen’s effect size d for different risk factors of patellofemoral instability in patients who suffered from patellar dislocation and healthy control subjects

Table 1.1						
**NWB - CONTROL GROUP VS. PATIENT GROUP**						
		**ISI**	**CDI**	**PTI**	**PTA-M**	**PTA-S**
**NWB CONTROLS**	**mean**	1.06	1.01	0.49	7.62	9.27
	**SD**	0.19	0.13	0.18	4.60	5.26
**NWB PATIENTS**	**mean**	1.23	1.16	0.49	14.00	16.34
	**SD**	0.22	0.15	0.16	7.54	7.84
	**difference**	0.17	0.15	−0.01	6.38	7.07
	***p*-value**	**< 0.001**	**< 0.001**	0.8305	**< 0.001**	**< 0.001**
	**POWER**	0.93	0.99	–	0.99	0.99
	**cohen’s d**	0.85	1.04	−0.04	1.05	1.08
**WB - CONTROL GROUP VS. PATIENT GROUP**						
		**ISI**	**CDI**	**PTI**	**PTA-M**	**PTA-S**
**WB CONTROLS**	**mean**	1.13	1.10	0.47	7.57	9.59
	**SD**	0.24	0.16	0.18	5.17	5.47
**WB PATIENTS**	**mean**	1.23	1.21	0.45	15.97	18.54
	**SD**	0.20	0.17	0.19	9.10	9.43
	**difference**	0.10	0.12	−0.02	8.40	8.95
	***p*-value**	**0.0206**	**0.0096**	0.6043	**< 0.001**	**< 0.001**
	**POWER**	0.47	0.81	–	0.99	1.00
	**cohen’s d**	0.46	0.69	−0.10	1.18	1.20
Table 1.2						
**CONTROL GROUP – NWB VS. WB**						
		**ISI**	**CDI**	**PTI**	**PTA-M**	**PTA-S**
**NWB CONTROLS**	**mean**	1.06	1.01	0.49	7.62	9.27
	**SD**	0.19	0.13	0.18	4.60	5.26
**WB CONTROLS**	**mean**	1.13	1.10	0.47	7.57	9.59
	**SD**	0.24	0.16	0.18	5.17	5.47
	**difference**	0.07	0.09	−0.02	−0.05	0.32
	***p*-value**	0.1372	**0.0164**	0.4992	0.8542	0.8584
	**POWER**	–	0.66	–	–	–
	**cohen’s d**	0.35	0.58	−0.14	−0.01	0.06
**PATIENT GROUP – NWB VS. WB**						
		**ISI**	**CDI**	**PTI**	**PTA-M**	**PTA-S**
**NWB PATIENTS**	**mean**	1.23	1.16	0.49	14.00	16.34
	**SD**	0.22	0.15	0.16	7.54	7.84
**WB PATIENTS**	**mean**	1.23	1.21	0.45	15.97	18.54
	**SD**	0.20	0.17	0.19	9.10	9.43
	**difference**	0.00	0.05	−0.04	1.97	2.20
	***p*-value**	0.9485	0.1657	0.2332	0.4629	0.4313
	**POWER**	–	–	–	–	–
	**cohen’s d**	0.02	0.31	−0.22	0.24	0.25

*WB* Weight bearing, *NWB* Non weight-bearing, *ISI* Insall-Salvati-Index, *CDI* Caton-Deschamps-Index, *PTI* Patellotrochlear Index, *PTA-M* modified Fulkerson’s patellar tilt, *PTA-S* Sasaki’s patellar tilt. Significant *p*-values (*p* < 0.05) marked in dark grey. *POWER* Post-hoc power value for significant results

Inter-class-correlation coefficient (ICC) for inter- and intra-observer reliability of our measurements was very good to excellent (Table 2).

**Table 2:** Intra- and Inter-Observer-Reliability, calculated by Intra-Class-Correlation, figures in per cent (%)(t_1_: first measurement 1, t_2_: repeated measurement 2; E_1_: Examiner 1, E_2_: Examiner 2)

		ISI	CDI	PTI	PTA-M	PTA-S
Intra-Observer-ReliaBility (t_1_ vs t_2_)	supine	96.2	84.4	97.2	87.3	85.1
	upright	90.9	88.1	96.3	91.2	89.9
Inter-Observer-reliability (E_1_ vs E_2_)	supine	95.7	76.0	95.3	86.3	85.7
	upright	86.5	79.0	92.5	89.9	89.0

*ISI* Insall-Salvati-Index, *CDI* Caton-Deschamps-Index, *PTI* Patellotrochlear Index, *PTA-M* Modified Fulkerson’s patellar tilt angle, *PTA-S* Sasakis patellar tilt angle

With exclusion of the PTI, all assessed indices showed significant differences between the patient group and the control group in supine as well as in upright position.

Comparing pairwise all measured indices detected in supine and weight bearing position within the two groups showed no significance between the two different MRI settings. Only the values of the Caton-Deschamps-Index of the control group showed a significant difference respecting the MRI position (*p* = 0.0164). For the patient group, ISI and CDI showed borderline values already in supine position (ISI 1.23 ± 0.22, CDI 1.16 ± 0.15) without further significant worsening in the weight-bearing position.

Regarding the values of patellar tilting, the measured PTA-M within the patient group showed an elevation from supine to weight bearing position (14.00 ± 7.54° to 15.97 ± 9.10°) whereas it stayed nearly the same for the healthy controls (7.62 ± 4.60° supine versus 7.57 ± 5.17° weight bearing).

Similar values were assessed for the PTA-S (16.34 ± 7.84° supine to 18.54 ± 9.43° upright in the patient group compared to 9.27 ± 5.26° supine to 9.59 ± 5.47° upright in the control group). Still, all named results failed to show statistical significance.

Cohen’s d showed small to medium effects in the patient group between supine and weight bearing positions for PTA-M (0.24) and PTA-S (0.25), whereas the control group showed no such effect (PTA-M 0.01, PTA-S 0.06). Concerning patellar height, greater effects were observed in the control group than in the patient group for ISI (0.35 vs. 0.02) and CDI (0.58 vs. 0.31) in regard to the change of the patient’s position.

PTI showed small to medium Cohens’s effect changes in both groups (− 0.14 in the control group, − 0.22 in the patient group).

## Discussion

The most important finding of the presented study is, that in comparison to supine MRI investigation, particular risk indices for patellofemoral instability in affected patients show divergent results under the setting of a WB-MRI and 20° flexion angle of the knee.

Though - in contrast to our hypothesis - these results failed to show clear significant statistical differences between weight bearing and non-weight bearing MRI, the calculation of Cohen’s effect size (d) confirmed small to medium effects especially for patellar tilt values (PTA-S, PTA-M) within the patient group. This implicates at least a moderate clinical and practical relevance for the use of additional axial weight-bearing MRI planes in an early knee flexion angle of 20° in particular patients. Hence the previously described 20% of patients suffering from chronic patellofemoral problems and having inconspicuous risk values on standard MRI [4] might benefit from the additional performance of a WB-MRI in 20° flexion angle of the knee.

Not least, another important finding of our study is that the selected 20° flexion angle of the knee does not seem to be detrimental. Nearly all indices (except of PTI) showed significant differences between the control group and the patient group in supine as well as in weightbearing MRI. This suggests that MR imaging conducted in the chosen flexion angle of the knee seems to at least demonstrate patellofemoral maltracking in affected patients correctly when considered from a qualitative perspective.

However, the comparative and quantitative expressiveness of patellofemoral maltracking measured in 20° flexion angle of the knee instead of in full extension is limited. This is due to the circumstance that our study did not include an equivalent control group with positioning of the knee in full extension during MRI investigation. Instead, our intention was to assure an (additional) investigation protocol that could be implemented in daily routine and therefore one knee flexion angle was selected. With a total duration of approximately 15 min required for the MRI investigation, the selected protocol seems easily applicable in daily routine.

We assumed that the quantity of contributing factors of patellofemoral maltracking might be displayed more realistically, thus facilitating a more accurate diagnostic understanding in particular cases.

Also, other authors [1] concluded that the extent of surgical options, e.g. the amount of transfer of the tibial tubercle, should be defined with caution when solely established on the findings of standard MRI in full knee extension. Due to an incongruity between the results of the supine and weightbearing MRI their findings confirmed a decrease in pathological values in correspondence with higher knee flexion angles [1].

In their similar study setup Becher et al. [1] studied 16 patients with patellofemoral instability under weight-bearing and non-weight-bearing circumstances in 0°, 15°, 30° and 45° flexion angles of the knee. Also measuring patellar height indices (ISI, CDI, PTI) as well as patellar tilt, bisect offset and Tuberosity Trochlear Groove Distance (TTTG), their most important finding was a significant correlation effect between WB-MRI and the knee flexion angle especially in extension and near flexion angles.

In summary, they concluded that the performance of a WB-MRI in different knee flexion angles affects patellofemoral MRI indices which show a significant increase at full extension of the knee. Consequently, in order to balance the necessity and extent of therapeutic options, they suggested an MRI investigation under both weight-bearing and non-weight-bearing circumstances in full extension as well as flexion of the knee. However, the total amount of their MRI investigation protocol was not mentioned and can only be assumed to have lasted longer than the 15 min scheduled for our protocol. Therefore, despite scientific coherence an elaborate investigation as such will probably fail to establish itself in daily clinical routine due to required time and economic aspects.

In the patient group quantitative values determined sagittal risk factors (ISI, CDI, PTI) displayed unaltered highly normal or pathological values of patellar height, irrespective of the patient’s positioning during MRI. This seems somehow self-explanatory for ISI and CD as ratio-relevant parameters like the length of the patella, the length of the patella tendon and the diagonal length of the patella are not expected to show a relevant change even under contraction of the extensor apparatus. In contrast, the unchanged PTI of our results reflects that the patella has not yet entered into the trochlear groove at the 20° flexion angle of the knee. If not guided by the trochlear groove the dynamic displacement of the patella during transition from knee extension into bony constraint might be displayed best in 20° flexion of the knee.

Despite not listing all singular values the graphically displayed results of Becher et al. [1] also reveal constant patellar height indices (ISI, CDI) beyond 15°. Of course, higher values were assessed for the PTI in 30° and 45° flexion angles reflecting an increased guiding of the patella by the trochlear groove. Again, in terms of practicability it seems questionable that these higher knee flexion angles are necessary and beneficial in daily routine as the results confirm that the relevant and significant change in the measured indices seems to occur within the first 15° to 20° knee flexion.

Though not significantly, greater values were assessed for the patella tilt in knee extension under weight-bearing than in the supine MRI investigation in the named study [1]. Here it was shown that the patellar tilt value decreases along with the increase in the knee flexion angle, revealing the greatest decline within the first 15° and then subsequently “slowing down” the index correction in 30° and 45° knee flexion angles. Additionally, there is a relevant loss of difference between the results of WB-MRI and the MRI performed in supine positioning of the patient with increase in the knee flexion angle.

The results of the present study also showed higher values of the patellar tilt (PTA-S + PTA-M) under weightbearing conditions at 20° knee flexion. Though the changes failed to reveal significance they at least showed a minor to moderate effect size in regard to Cohen’s d (0.24 + 0.25).

Consequently, considering that significance and effectiveness between weightbearing and supine MRI rather decreases with increasing knee flexion angles [1], and presuming that the sole performance of a non-WB MRI in knee extension might display overly negative indicator values [1, 12] conducting further axial WB-MRI planes at a 20° flexion angle of the knee could represent the “happy medium” offering beneficial information in selected patients. Not least, this approach would allow a suitable setup for daily routine as these single MRI planes would not require a notable increase in time or cost.

There were some limitations of our presented study. Most important, a control group with a 0° flexion angle of the knee during the performed MRI examinations was not included in our study. In accordance with the studies of Becher et al. [1, 7] and in retrospect it would have been beneficial to have had comparable values for knee flexion angles of 0° and 20° in order to emphasize the necessity of both angles for therapeutic conclusions. However, the results of the underlying studies [1, 7] in combination with our results which included a remarkably higher number of subjects, at least allow a fundamental discussion about the benefit of various flexion angles for therapeutic decisions. Not least, patients in our study were not aligned evenly by sex. However, the authors would not expect relevant deviations from the presented results just because of an equalization of this imbalance.

Secondly, as previous authors [1, 10] have already stated MRI remains a static, non-dynamic imaging technique. Joint kinematics in dynamic situations still have to be respected when adequately considering underlying pathologies. Thus, in clinical decision making the results of our study as well as the drawn conclusions must be considered in combination of functional and dynamic aspects.

A further relevant and debatable point is that we abstained from evaluating the TTTG-distance. This is mainly reasoned because we intended to investigate parameters with involvement of the patella. Furthermore, previous studies offer the common conclusion that the TTTG significantly depends on the flexion of the knee showing higher values in extension due to the “screw home” mechanism [1, 23]. Therefore, to the authors the TTTG recently has lost some of its value in favor of the TTPCL (Tibial Tuberosity – Posterior Cruciate Ligament) which is not affected by the position of the knee and due to this is regarded to be more reliable during pre-operative planning.

In accordance with previous studies, performing a study in which the individuals investigating the MRI examinations are blinded is not possible. Visible signs of instability or recurrent dislocations such as a rupture of the MPFL or lateral bone bruise obviated a blinded assignment due to the apparent association of the participant to either the one or the other group.

Not least, despite the known difference of the index values between the control group and the patient group statistical significance between the supine and WB-MRI was not achieved. Post-Hoc analysis revealed that higher cohort numbers might balance this weakness. Nonetheless, this makes the necessity of WB-MRI of the knee in early flexion angles in itself appear questionable. Nevertheless, respecting a moderate effect size for particular indices we still suggest the presented protocol for more accurate diagnostic understanding in selected cases.

## Conclusion

Though the results of this study failed to show statistically significant differences of patellofemoral risk factors between supine and upright MRI for sagittal parameters in 20° knee flexion, Cohen’s d showed a clinical effect of additional upright MRI regarding the accentuation of differences between healthy and pathologically unstable patellofemoral joints. Instead of sagittal parameters (ISI, CD, PTI), axial parameters (PTA-M + PTA-S) revealed considerable differences and should therefore especially be considered when it comes to therapeutic decision making. Regarding our findings as well as the conclusions of previous studies [4, 6, 7] which could not reveal significant effects of WB-MRI in 30° and 45° knee flexion angles in comparison to lower flexion angles, further studies could focus on the conduction of additional axial MRI planes in solely 20° flexion of the knee.

Nevertheless, new thresholds for pathological values and relative changes between the different patient’s different positions need to be evaluated in order to maximize the information for clinical assessment and therefore to possibly improve the outcome for patient and therapist.

## Data Availability

The datasets used and analysed during the current study are available from the corresponding author upon reasonable request.

## Abbreviations

ACL: Anterior cruciate ligament
MCL: Medical collateral ligament
TR: Time to repeat
TE: Time to echo
FOV: Field of view
PD: Proton density
ST: Slite thickness
MPFL: Medial patellofemoral ligament

## References

[1] Becher C, Fleischer B, Rase M (2017). Effects of upright weight bearing and the knee flexion angle on patellofemoral indices using magnetic resonance imaging in patients with patellofemoral instability. Knee Surg Traumatol Arthrosc.

[2] Draper CE, Besier TF, Fredericson M (2011). Differences in patellofemoral kinematics between weight-bearing and non weight-bearing conditions in patients with patellofemoral pain. J Orthop Res.

[3] Fulkerson JP (2002). Diagnosis and treatment of patients with patellofemoral pain. Am J Sports Med.

[4] Mariani S, La Marra A, Arrigoni F (2015). Dynamic measurement of patellofemoral joint alignment using weight-bearing magnetic resonance imaging (WB-MRI). Eur J Radiol.

[5] Fredericson M, Yoon K (2006). Physical examination and patellofemoral pain syndrome. Am J Phys Med Rehabil.

[6] Balcarek P, Oberthür S, Hopfensitz S (2014). Which patella is likely to redislocate?. Knee Surg Traumatol Arthrosc.

[7] Becher C, Schumacher T, Fleischer B (2015). The effects of a dynamic patellar realignment brace on disease determinants for patellofemoral instability in upright weight-bearing condition. J Orthop Surg Res.

[8] Hingelbaum S, Best R, Huth J (2014). The TT-TG index: a knee size adjusted measure method to determine the TTTG distance. Knee Surg Traumatol Arthrosc.

[9] Liebensteiner MC, Dirisamer F, Balcarek P (2017). Guidelines for treatment of lateral patella dislocations in skeletally mature patients. Am J Orthop.

[10] Bruno F, Barile A, Arrigoni F (2018). Weight-bearing MRI of the knee: a review of adavantages and limits. Acta Biomed.

[11] Pal S, Besier TF, Draper CE (2012). Patellar tilt correlates with vastus lateralis: vastus medialis activation ratio in maltracking patellofemoral pain patients. J Orthop Res.

[12] Senavongse W, Amis AA (2005). The effects of articular, retinacular or muscular deficiencies on patellofemoral joint stability: a biomechanical study in vitro. J Bone Joint Surg Br.

[13] Hirschmann A, Buck FM, Herschel R (2017). Upright weight-bearing CT of the knee during flexion: changes of the patellofemoral and tibiofemoral articulations between 0° and 120°. Knee Surg Sports Traumatol Arthrosc.

[14] Hirschmann A, Buck FM, Fucentese SF (2015). Upright CT of the knee: the effect of weight-bearing on joint aligment. Eur Radiol.

[15] Insall J, Salvati E (1971). Patella position in the normal knee joint. Radiology..

[16] Caton J, Deschamps G, Chambat P (1982). Patella infera. Apropos of 128 cases. Rev Chir Orthop Reparatrice Appar Mot.

[17] Biedert RM, Albrecht S (2006). The patellotrochlear index: a new index for assessing patellar height. Knee Surg Traumatol Arthrosc.

[18] Lin YF, Jan MH, Lin DH (2008). Different effects of femoral and tibial rotation on the different measurements of patella tilting: an axial computed tomography study. J Orthop Surg Res.

[19] Sasaki T, Yagi T (1986). Subluxation of the patella: investigation by computed tomography. Int Orthop.

[20] Cohen J, Wolman BB (1965). Some statistical issues in psychological research. Handbook of Clinical Psychology.

[21] Sullivan GM, Feinn R (2012). Using effect size – or why the P value is not enough. J Grad Med Educ.

[22] Cicchetti DV (1994). Multiple comparison methods: establishing guidelines for their valid application in neuropsychological research. J Clin Exp Neuropsychol.

[23] Izadpanah K, Weitzel E, Vicari M (2014). Influence of knee flexion angle and weight bearing on the Tibial tuberosity-trochlear groove (TTTG) distance for evaluation of patellofemoral alignment. Knee Surg Traumatol Arthrosc.

